# Hypertensive patients' use of blood pressure monitors stationed in pharmacies and other locations: a cross-sectional mail survey

**DOI:** 10.1186/1472-6963-8-216

**Published:** 2008-10-22

**Authors:** Anthony J Viera, Lauren W Cohen, C Madeline Mitchell, Philip D Sloane

**Affiliations:** 1 Department of Family Medicine, UNC Chapel Hill, USA; 2 Cecil G. Sheps Center for Health Services Research, UNC Chapel Hill, USA

## Abstract

**Background:**

Blood pressure (BP) monitors are commonly stationed in public places such as pharmacies, but it is uncertain how many people with hypertension currently use them. We sought to estimate the proportion of hypertensive patients who use these types of monitors and examine whether use varies by demographic or health characteristics.

**Methods:**

We conducted a cross-sectional mail survey of hypertensive adults enrolled in a practice based research network of 24 primary care practices throughout the state of North Carolina. We analyzed results using descriptive statistics and examined bivariate associations using chi-square and independent associations using logistic regression.

**Results:**

We received 530 questionnaires (76% response rate). Of 333 respondents (63%) who reported checking their BP in locations other than their doctor's office or home, 66% reported using a monitor stationed in a pharmacy. Younger patients more commonly reported using pharmacy monitors (48% among those < 45 years vs 35% of those over 65, p = 0.04). Blacks reported using them more commonly than whites (48% vs 39%, p = 0.03); and high school graduates more often than those with at least some college (50% vs 37%, p = 0.02). In multivariate analysis, younger age (aOR 1.49; 95% CI 1.00–2.21 for those age 45 to 65 years vs those > 65 years old) and high school education (aOR 1.74; 95% CI 1.13–2.58) were associated with use of pharmacy-stationed monitors, but Black race was not. Patients with diabetes, heart disease, or stroke were not more likely to use pharmacy-stationed monitors.

**Conclusion:**

Hypertensive patients' use of BP monitors located in pharmacies is common. Younger patients, Blacks, and those with high school education were slightly more likely to report using them. Because use of these monitors is so common, efforts to ensure their accuracy are important.

## Background

Publicly available blood pressure (BP) monitors (such as those located in pharmacies, retail stores, community centers, and fitness centers) could be an attractive BP monitoring option for people who do not have the resources to purchase their own BP monitor or for people who need to check their BP relatively infrequently outside their health care visits. However, the few studies evaluating these monitors have shown them to be inaccurate compared to readings taken manually using mercury sphygmomanometry [1-5].

According to the standards set forth by the Association for the Advancement of Medical Instrumentation (AAMI), the mean difference (using a standard protocol) between a test monitor's readings (systolic and diastolic treated separately) and readings made using mercury sphygmomanometry must be within ± 5 mm Hg with a standard deviation of no more than 8 mm Hg [6]. The AAMI standard states that if the gold standard for BP measurement is auscultation, then at least 85 subjects must be tested and at least 255 individual readings recorded. If intra-arterial pressure is the gold standard, then at least 150 individual readings are necessary. We found only one report of a public-use device that met the AAMI standard [7].

Several studies (most evaluating the Vita-Stat models) have found public-use, community-based monitors to not meet acceptable standards of accuracy and reliability [1-5]. While mean differences between mercury sphygmomanometry and the community-based devices tended to be small (e.g., 4.4 mm Hg difference in systolic and 1.0 mm Hg difference in diastolic) the 95% confidence intervals around these estimates were quite large (e.g., 14 mm Hg below or 23 mm Hg above the systolic reading taken with a mercury manometer)[2].

Correct cuff size is an important factor when measuring BP. A cuff that is too small will give artificially high BP values while a cuff that is too large may underestimate BP. Currently, most pharmacy-based units have one cuff for arms of all sizes. Unless cuffs inflate in a specific way to ensure that the proper functional cuff size is used for patients with various arm sizes, measurement accuracy can be affected. One group of investigators evaluated monitors stationed in 25 randomly selected community pharmacies using the same three volunteers (small, medium, and large arm sizes), and the same member of the research team took all the mercury readings [3]. Average differences between mercury manometry and the public-use monitors varied from within about 8–10 mm Hg for people with small and large arms to within 2–3 mm Hg for people with medium-sized arms [3]. Again there were large average standard deviations of the mean differences (≥ 8 mm Hg) [3]. While the accuracy was best in people with medium-sized arms, the reliability in that group was the poorest.

Despite these concerns about the accuracy and reliability of public BP monitors, they are commonplace. In 2004, there were an estimated 30,000 public-use BP monitors stationed in pharmacies and worksites in the United States [7]. These monitors are undoubtedly being used by patients, but the number of patients using these devices is not clear. Another consideration is that with the advent of more affordable, technologically-improved home BP monitors, their use is very common among patients with hypertension. In our recent study we found that more than 40% of hypertensive patients use a home BP monitor [8]. Given this finding, the use of pharmacy monitors may have declined in recent years. The purpose of this study was therefore to determine the prevalence of hypertensive patient's use of BP monitors stationed in pharmacies and other locations. We also sought to determine whether use of these types of monitors varied by hypertensive patients' demographic and health status characteristics.

## Methods

### Overall Design

This study was a cross-sectional survey of adult patients seen in practices within the North Carolina Family Medicine Research Network. The 24 practices in the network were selected to represent the geographic regions and ethnic diversity of the state of North Carolina. Through this practice based research network (PBRN), a cohort of patients was developed for the purpose of facilitating research on health care problems commonly addressed in primary care settings [9]. A complete description of the network's scope and design is presented elsewhere [9].

For initial enrollment in the PBRN cohort, all patients 18 years and older who presented for a scheduled office visit during a four-week period were approached for participation. Of those, 64% agreed to participate [9]. Subjects who agreed to participate were given a four-page enrollment questionnaire containing standardized questions on self-reported chronic conditions and health habits as well as demographic items. Subjects were asked if they were willing to be contacted for future studies, and 82% granted such permission [9]. The current study surveyed a sample of those individuals who completed the enrollment survey. This study was approved by the Biomedical Institutional Review Board of the University of North Carolina at Chapel Hill School of Medicine.

### Sample

In the updated year 2004 to 2005 cohort (n = 2720) of patients enrolled in the PBRN, 1088 patients indicated on their enrollment questionnaire that they had high BP. From these 1088 potential subjects we drew a computer-generated random sample of 700 individuals stratified by race (446 whites and 254 Blacks).

### Survey Instrument and Variables

The primary intent of the BP monitoring questionnaire was to examine the use of home blood pressure monitoring (HBPM) by hypertensive patients (results published previously [8,10]); therefore our sample size was based on this outcome. We anticipated a 60% response rate, or 420 returned questionnaires, which would allow precision of within 5%, assuming the proportion that used HBPM was 10%. After an initial drafting and revisions of the questionnaire, the final product was guided by a focus group in which clinic nurses and medical assistants were queried regarding their perceptions of patients' performance of out-of-office BP monitoring gleaned from their interactions with patients. The questions were also assessed for clarity and readability during this focus group.

In addition to estimating patients' use of HBPM, we also wanted to explore patients' use of other methods of out-of-office monitoring. Therefore, we also included the partially open-ended question, "Is there a place you go other than your doctor's office to check your blood pressure?" Those who responded "Yes" indicated whether they went to a pharmacy (including those located within a larger retail store), fitness/community center, or other location (with write-in option). Write-in options were re-categorized into one of the designated categories as appropriate, and we created the additional category of "other medical facility" based on several responses. Remaining options were categorized as "other." Respondents could also indicate whether they had a friend or relative check their BP, and respondents could choose more than one option.

For incorporating independent variables of interest, the questionnaire data were linked to the variables already maintained on respondents (e.g., demographics, other health conditions and behaviors). Cardiovascular conditions and health behaviors were based on respondents' previous answers to items on the enrollment questionnaire. For example, the presence of heart disease was based on the respondent's answer to the item: "Please indicate whether or not you have had any of these problems within the past ten years." This was followed by a list of items with the option to indicate "Yes" or "No" for each condition. "Heart disease" was one of the items listed, as was "diabetes" and "stroke or mini-stroke." Smoking status was based on answer (Yes/No) to the question, "Do you smoke at least one cigarette per day?" Age, self-reported health status, and education level were each divided into three categories. Body mass index (BMI) was computed based on self-reported height and weight and divided into three categories.

### Survey Administration and Data Management

The survey was mailed with a cover letter and postage-paid return envelope to the 700 potential respondents using the most recent address in the PBRN database. As a small token of appreciation, a $1 bill was attached to the questionnaire. A reminder/thank-you postcard was mailed to all 700 potential respondents one week later. A second questionnaire was mailed to nonrespondents three weeks later. Six weeks after the second mailing, attempts were made to contact remaining non-respondents by telephone. Those contacted were offered completion of the questionnaire via telephone or a third mailing. Upon receipt of completed surveys, the data were double-entered into a Microsoft Access database. Once all survey data were entered, the data were imported into standard statistical software, compared for accuracy, checked for logical errors, and cleaned and compiled.

### Analysis

After performing a series of exploratory analyses to insure the integrity of the data, we determined percentages of respondents within categories of the independent variables. We then determined proportion of respondents who reported using monitors stationed in pharmacies (including pharmacies within larger retail stores), fitness/community centers, other medical facilities, and other locations. We determined the proportion who indicated that they had a friend or relative check their BP. We examined bivariate associations of the independent variables with reports of using a pharmacy-stationed monitor and tested for significance using Pearson chi-square. Lastly, we examined the independent effects of these associations by fitting a multivariable logistic regression model. All analyses were performed using Stata 8.1 statistical software (StataCorp, College Station, TX).

## Results

Of the 700 questionnaires mailed, 25 were undeliverable. There were 15 mail refusals, and 11 recipients were ineligible (9 were deceased; 1 was incarcerated; 1 did not have hypertension). Following the first mailing, 433 questionnaires were returned completed. An additional 31 questionnaires were returned completed after the second mailing. During the period of telephone follow-up, an additional 66 questionnaires were completed for a conservative, unadjusted response rate of 76% (530/700). After excluding ineligibles (n = 11) and undeliverables (n = 25) the response rate was 80% (530/664).

Respondents had a mean age of 59.6 ± 13.7 years. The majority were female (68.5%), white (67.5%), overweight or obese (86.1%), did not smoke (79.2%), and had at least a high school education (74.4%) (Table 1). More than one-fourth (26.0%) reported having heart disease; one-third had diabetes, and 10.3% reported a history of a stroke or mini-stroke (transient ischemic attack, or TIA). Nonrespondents were disproportionately younger, Black, and male.

**Table 1:** Characteristics of survey respondents (n = 530)

	**Percent (%)**
**Age category (years)**	
**< 45**	14.2
**45 to 65**	50.4
**> 65**	35.5

**Male**	31.5

**Race**	
**Black**	32.5
**White**	67.5

**Education**	

**< High school graduate**	25.6
**High school graduate**	32.5
**Some college or more**	41.9

**Reports little/no exercise**	42.3

**Body mass index**	
**Normal (< 25 kg/m**^2^**)**	14.0
**Overweight (25 to 30 kg/m**^2^**)**	30.8
**Obese (> 30 kg/m**^2^**)**	55.3

**Self reported health**	
**Excellent or very good**	21.2
**Good**	39.5
**Fair or poor**	39.3

**Current smoker**	20.8

**Other cardiovascular conditions**	
**Heart disease**	26.0
**Diabetes**	32.6
**Stroke or mini-stroke**	10.3

Overall, 62.8% (95% CI 58.7 to 67.0) of respondents reported checking their BP in a location besides their doctor's office or at home (Table 2). The most common location was a pharmacy, including pharmacies located in larger retail stores. Two-thirds of those who reported checking their BP in places other than their doctor's office or at home indicated they did so at a pharmacy. Most of the remainder (30%) selected "other place" for the location at which they checked their BP and did not use the option to write on the questionnaire the specific location. Many people had a friend or relative check their BP (16%). A small proportion reported checking their BP at another medical facility (5%), at their worksite (3%), or at a community or fitness center (2%).

**Table 2:** Proportion of respondents using blood pressure monitors stationed in pharmacies and other locations or sources of monitoring*

	**Among all respondents (n = 530)**		**Among the respondents who perform monitoring outside their doctor's office or home (n = 333)**	
	**Percent**	**95% confidence interval**	**Percent**	**95% confidence interval**

**Any location besides doctor's office or home**	62.8	58.7 – 67.0		
				
Pharmacy**	41.7	37.5 – 45.9	66.4	61.3 – 71.5
				
**Friend or relative checks BP**	8.9	6.4 – 11.3	14.1	10.4 – 17.9
				
**Other medical center (e.g., chiropractic office, health department**	2.8	1.4 – 4.2	4.5	2.3 – 6.7
				
**Worksite**	1.9	0.7 – 3.0	3.0	1.2 – 4.8
				
**Fitness center (e.g., YMCA) or community center (e.g., senior center)**	1.5	0.5 – 2.6	2.4	0.7 – 4.1
				
Other (e.g., fire department)***	18.9	15.5 – 22.2	30.0	25.1 – 35.0

*Respondents could select more than one location

** Includes pharmacy within larger retail stores, e.g., Wal-Mart or Target

*** Most respondents who selected "other" did not write in a specific location, prohibiting reclassification or sub-classification of this category

In bivariate analyses (Table 3), respondents older than 65 years of age were less likely to use pharmacy-stationed monitors (35% vs approximately 48% and 45% of those in each of other two age groups, p = 0.04). Race was also significant, with 48.3% of Blacks using pharmacy-stationed monitors compared to 38.6% of whites (p = 0.03). There was also a difference noted by education level: 36.5% of those with at least some college used a pharmacy-stationed monitor compared with 38% of people with less than high school education and 50% of high school graduates (p = 0.02). Respondents not currently using a home BP monitor were no more likely to use pharmacy-stationed monitors than those currently using a home BP monitor (44.5% vs 38.9%, p = 0.20). Patients with heart disease, diabetes, or history of stroke/TIA were no more likely to use pharmacy monitors than those without these conditions.

**Table 3:** Characteristics of hypertensive patients who use blood pressure monitors stationed in pharmacies

	**n/N**	**Percent**	**p-value**
**Age category (years)**			
**< 45**	36/75	48.0	0.04
**45 – 65**	120/267	44.9	
**> 65**	65/188	34.6	
**Sex**			
**Male**	75/167	44.9	0.31
**Female**	146/363	40.2	
**Race**			
**Black**	83/172	48.3	0.03
**White**	138/358	38.6	
**Education**			
**< High school graduate**	51/134	38.1	0.02
**High school graduate**	85/170	50.0	
**Some college or more**	80/219	36.5	
**Body mass index**			
**Normal (< 25 kg/m**^2^**)**	31/74	41.9	0.62
**Overweight (25–30 kg/m**^2^**)**	63/163	38.7	
**Obese (> 30 kg/m**^2^**)**	127/293	43.3	
**Self-reported health**			
**Excellent or very good**	44/112	39.3	0.72
**Good**	86/209	41.2	
**Fair or poor**	91/208	43.8	
**Current smoker**			
**Yes**	41/110	37.3	0.28
**No**	180/419	43.0	
**On BP medication(s)**			
**Yes**	202/484	41.8	0.32
**No**	19/43	44.2	
**Currently use a home BP monitor**			
**Yes**	88/226	38.9	0.20
**No**	133/299	44.5	
**Doctor recommended home BP monitoring**			
**Yes**	79/185	42.7	0.76
**No**	141/341	41.4	
**Heart disease**			
**Yes**	52/127	40.9	0.70
**No**	155/361	42.9	
**Diabetes**			
**Yes**	63/161	39.1	0.35
**No**	145/333	43.5	
**Stroke or mini-stroke**			
**Yes**	16/49	32.7	0.16
**No**	184/426	43.2	

In a logistic regression model (Table 4) that included age group, race, and education level, respondents in the 45 to 65 year age group were more likely than those older than 65 to use pharmacy stationed monitors (aOR 1.49, 95% CI 1.00 to 2.21). High school graduates were more likely than those with at least some college to use pharmacy stationed monitors (aOR 1.71, 95% CI 1.13 to 2.58). Black race was not significant in the multivariate model.

**Table 4:** Unadjusted and adjusted* odds ratios of characteristics associated with use of blood pressure monitors stationed in pharmacies

	**Unadjusted OR**	**95% CI**	**Adjusted OR**	**95% CI**
**Age category (years)**				
**< 45**	1.75	1.01 – 3.01	1.57	0.89 – 2.76
**45 – 65**	1.54	1.05 – 2.27	1.49	1.00 – 2.21
**> 65**	referent		referent	
**Race**				
**Black**	1.49	1.03 – 2.15	1.40	0.95 – 2.05
**White**	referent		referent	
**Education**				
**< High school graduate**	1.07	0.69 – 1.66	1.08	0.68 – 1.72
**High school graduate**	1.74	1.16 – 2.61	1.71	1.13 – 2.58
**Some college or more**	referent		referent	

*Based on logistic regression model adjusted for other characteristics in the table

## Discussion

Nearly two-thirds of hypertensive adults in this sample of patients from a primary care population reported checking their BP in locations other than their doctor's office or at home. As expected, the most commonly used monitors were those located in pharmacies. Our study did not assess use of these monitors by people who have not been diagnosed with hypertension, but such use is also likely to be very common. BP measurement devices in pharmacies and other retail stores are typically automatic oscillometric kiosk-type monitors. Since so many people use these types of monitors – either for monitoring or self-screening – efforts to ensure their accuracy are needed.

Given that older patients visit pharmacies more often than younger patients, we were surprised to find that younger patients were more likely to report using pharmacy monitors. In our earlier study of this same hypertensive population, 43% of respondents indicated that they use HBPM [8]. Patients 65 years and older were more likely than patients younger than 45 to use HBPM (47% vs 29%, p = 0.03) [8]. This difference may partially explain our finding in this study that older patients are less likely to monitor their BP using a pharmacy-stationed monitor, although we did not find that patients currently using HBPM in general were less likely to use pharmacy-stationed monitors. Older patients may also use pharmacy monitors less often because they make more frequent visits to their physicians' offices, and therefore do not perceive a need to monitor more frequently.

In bivariate analysis, Blacks were more likely than whites to use pharmacy-stationed monitors. This may reflect that hypertension is more common in Blacks and their BP is more difficult to control [11]. After adjustment for age group and education level, race was not significant. We also found that respondents with high school level of education were more likely to use pharmacy-stationed monitors than either those with less than high school education or more than high school education. The reasons for this are not clear.

We did not find that patients with comorbidities such as diabetes, heart disease, or stroke were more likely to use pharmacy-stationed monitors. One might suspect that patients at greater cardiovascular disease risk would monitor their BP status more frequently. More frequent HBPM use among these patients would support this notion. However, in our HBPM study, we only found a trend toward more frequent HBPM use among patients with a history of stroke/TIA [8]. We did not find more frequent HBPM use among patients with diabetes or heart disease [8]. One explanation may be that patients with additional conditions and risk factors such as these are visiting their physician's office more frequently and therefore not perceiving a need to monitor their BP at other times.

We suspected that we might find a higher proportion of respondents using pharmacy monitors if their doctor recommended that they perform home BP monitoring (e.g., if they did not want to or could not afford to purchase a home BP monitor). We also suspected that we would find fewer people using pharmacy-stationed monitors if they had their own home BP monitor. We found neither situation to be the case, however.

Despite their seemingly ubiquitous presence, there are few other published studies on the prevalence of use of public BP monitors. One prior cross-sectional study of adult primary care patients with hypertension found that 63% of respondents reported "having used an automatic blood pressure machine" located in a retail store [12]. This proportion is similar to what we found. Also similar to our finding, that study found that hypertensives who used public BP monitors were slightly younger. However, dissimilar was their finding that those who used public monitors had higher education levels. Perhaps the most important finding in their study was the range of responses people had after using public monitors: 28% sought treatment, 10% changed their medication, 30% reported increasing exercise, and 33% reported changing their diet [12]. Thus, there are two important reasons for assuring the accuracy of public-use BP monitors: a lot of people use them, and people make decisions based on the BP measurements from them.

### Limitations

One consideration is whether the PBRN cohort is representative of the population of North Carolina. To gain an appreciation of the overall representativeness of this sample, we made a comparison of the initial PBRN cohort enrollees with the Center for Disease Control's Behavioral Risk Factor Surveillance System survey, which is a random telephone sample of all adults in the state. The populations are quite comparable other than the higher enrollment rates for women and middle-aged persons, which are reflective of primary care practice in general [9].

For this study, we did not ask respondents to indicate how often they used pharmacy-stationed (or other publicly available) monitors. Of note, the prior study mentioned above found that people used them an average of 13 times in the course of a year [12]. We also did not examine whether patients' use of BP monitoring in locations other than their doctor's office or home was associated with better BP control.

Another limitation is possible selection bias resulting in a sample of people generally more interested in their health. Therefore, caution must be used in generalizing our findings. The actual proportion of hypertensive adults who currently use pharmacy and other store monitors may be higher or lower than estimated, depending on the degree to which the sample may differ from the overall primary care population.

## Conclusion

Use of BP monitors located in pharmacies and other public locations is common among hypertensive patients. Hypertensive patients who are younger, Black, and have a high school education tend to use pharmacy monitors more commonly. Clinicians caring for hypertensive patients should be aware that public-use BP devices are potentially valuable for monitoring of persons who cannot afford or who do not wish to use a home BP monitor. However, as is the case for other modalities of BP measurement (including those taken by home and office devices), a single reading from a public-use device will often be inaccurate. Patients therefore should be cautioned not to over-react to any single reading. Instead, they should be encouraged to have multiple BP measurements taken and to write these down for discussion with their physician. Finally, as the technology of automatic devices continues to improve, the role of public-use BP monitors may expand over time; however, the current evidence suggests that home BP monitoring with an appropriate sized cuff using devices of proven accuracy may be a better option for most patients [13-16].

## Competing interests

The authors declare that they have no competing interests.

## Authors' contributions

AJV conceived of the study, performed the analyses, and drafted the initial manuscript and revisions. LWC and CMM assisted in revision of conceptual components of the study; provided expertise and oversight in survey administration and data management, assistance with data files, assistance with coding; and provided important suggestions for revisions of the manuscript. PLS provided key intellectual contributions to the design and execution of the NC Family Medicine Research Network itself, to this study, and to the manuscript. All authors read and approved the final manuscript.

## Pre-publication history

The pre-publication history for this paper can be accessed here:


